# 5-HTR_2A_ and 5-HTR_3A_ but not 5-HTR_1A_ antagonism impairs the cross-modal reactivation of deprived visual cortex in adulthood

**DOI:** 10.1186/s13041-018-0404-5

**Published:** 2018-11-06

**Authors:** Nathalie Lombaert, Maroussia Hennes, Sara Gilissen, Giel Schevenels, Laetitia Aerts, Ria Vanlaer, Lieve Geenen, Ann Van Eeckhaut, Ilse Smolders, Julie Nys, Lutgarde Arckens

**Affiliations:** 1 Laboratory of Neuroplasticity and Neuroproteomics, Katholieke Universiteit Leuven, Naamsestraat 59, Box 2467, B-3000 Leuven, Belgium; 2 Department of Pharmaceutical Chemistry, Drug Analysis and Drug Information, Center for Neurosciences (C4N), Vrije Universiteit Brussel, Laarbeeklaan 103, 1090 Brussels, Belgium; 3 Present Address: Laboratory of Synapse Biology, VIB-KU Leuven Center for Brain and Disease Research, O&N IV, Herestraat 49, box 602, B-3000 Leuven, Belgium

**Keywords:** Brain plasticity, Visual cortex, Adult mice, Monocular enucleation, Neuromodulator, Serotonin

## Abstract

Visual cortical areas show enhanced tactile responses in blind individuals, resulting in improved behavioral performance. Induction of unilateral vision loss in adult mice, by monocular enucleation (ME), is a validated model for such cross-modal brain plasticity. A delayed whisker-driven take-over of the medial monocular zone of the visual cortex is preceded by so-called unimodal plasticity, involving the potentiation of the spared-eye inputs in the binocular cortical territory. Full reactivation of the sensory-deprived contralateral visual cortex is accomplished by 7 weeks post-injury. Serotonin (5-HT) is known to modulate sensory information processing and integration, but its impact on cortical reorganization after sensory loss, remains largely unexplored. To address this issue, we assessed the involvement of 5-HT in ME-induced cross-modal plasticity and the 5-HT receptor (5-HTR) subtype used. We first focused on establishing the impact of ME on the total 5-HT concentration measured in the visual cortex and in the somatosensory barrel field. Next, the changes in expression as a function of post-ME recovery time of the monoamine transporter 2 (vMAT2), which loads 5-HT into presynaptic vesicles, and of the 5-HTR_1A_ and 5-HTR_3A_ were assessed, in order to link these temporal expression profiles to the different types of cortical plasticity induced by ME. In order to accurately pinpoint which 5-HTR exactly mediates ME-induced cross-modal plasticity, we pharmacologically antagonized the 5-HTR_1A_, 5-HTR_2A_ and 5-HTR_3A_ subtypes. This study reveals brain region-specific alterations in total 5-HT concentration, time-dependent modulations in vMAT2, 5-HTR_1A_ and 5-HTR_3A_ protein expression and 5-HTR antagonist-specific effects on the post-ME plasticity phenomena. Together, our results confirm a role for 5-HTR_1A_ in the early phase of binocular visual cortex plasticity and suggest an involvement of 5-HTR_2A_ and 5-HTR_3A_ but not 5-HTR_1A_ during the late cross-modal recruitment of the medial monocular visual cortex. These insights contribute to the general understanding of 5-HT function in cortical plasticity and may encourage the search for improved rehabilitation strategies to compensate for sensory loss.

## Introduction

Even though the mammalian brain is most susceptible to changes in sensory inputs during so-called critical periods early in life [1–4], it retains the intrinsic capacity to recover from sensory deprivation well into adulthood [1, 5–7]. In human patients, for example, late-onset vision loss triggers two types of cross-modal brain plasticity [8]: ‘compensatory plasticity’ is the result of experience-dependent refinement of the spared senses, whereas ‘cross-modal recruitment’ involves the take-over of the deprived visual cortical territory for the active processing of non-visual information, leading to enhanced sound localization abilities or improved tactile acuity as with Braille reading [9–13].

**Fig. 1:**
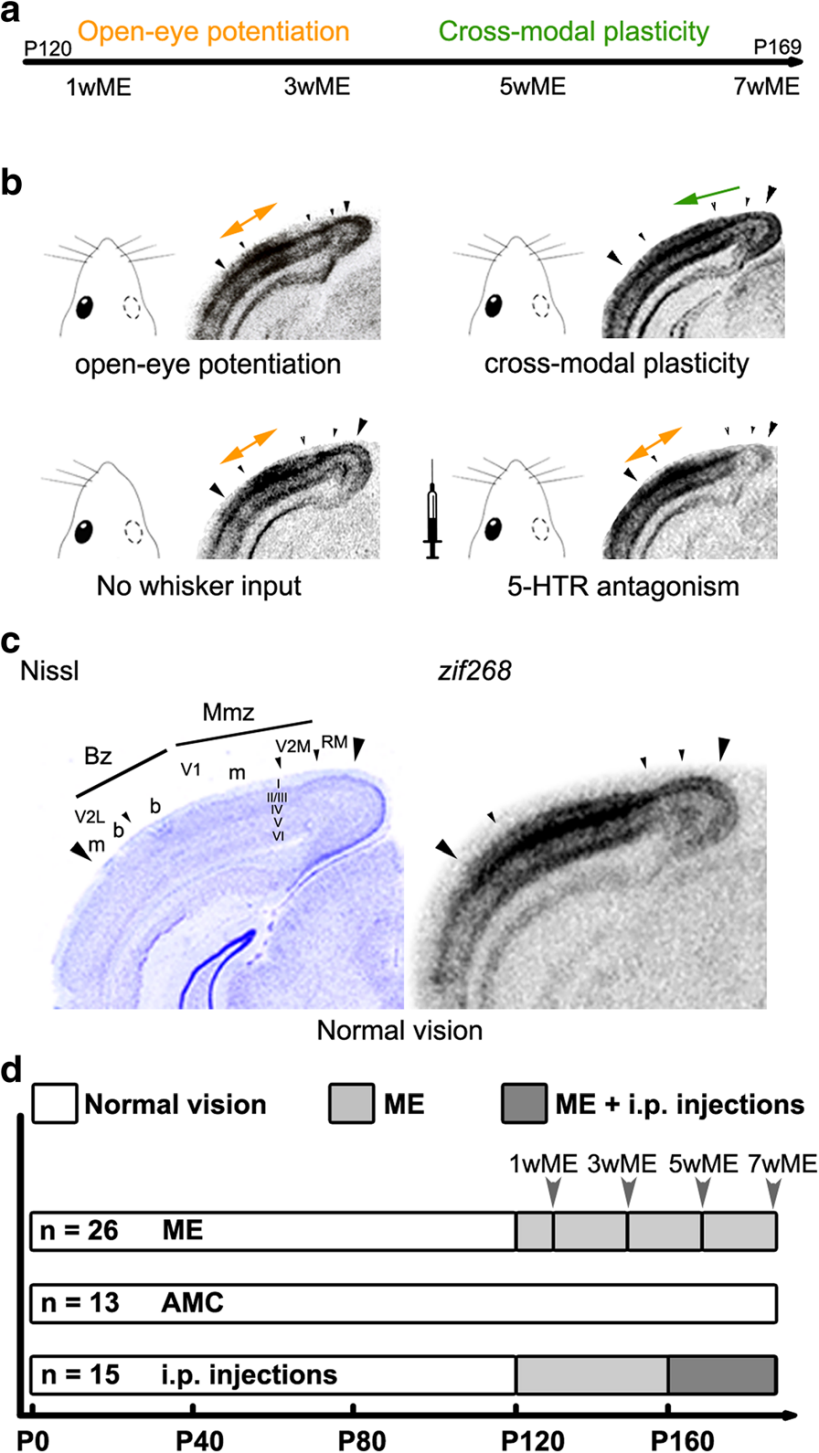
Schematic overview of the different cortical plasticity phases occurring in the adult ME-mouse model and illustration of the experimental setup. **a** Timeline indicating the early unimodal plasticity phase (week 1–3 post-ME) and the subsequent cross-modal plasticity phase (week 4–7 post-ME). **b** Overview of the ISH *zif268*-based reactivation pattern of the uni-and cross-modal plasticity phase in adult mice upon ME. Whisker deprivation and 5-HTR antagonism specifically suppress reactivation of the Mmz. **c** Illustration of the anatomical delineation of the visual cortex regions on a Nissl-stained coronal section which are then overlaid on the matching ISH *zif268* section. The delineated visual cortical areas are indicated between large arrowheads: V2L, V1, V2M and RM with the distinction between monocular (m) and binocular (b) segments. The binocular zone (Bz) comprises V2Lb-V1b while the medial monocular zone (Mmz) includes V1m-V2M. The different cortical layers are indicated with Roman numbers: I-VI. **d** All animals had normal vision up to the age of P120 or in case of the non-deprived age-matched control mice (AMC), up to P169 (white bars). Mice that underwent monocular enucleation (ME, light gray bars) at P120 recovered under standard housing conditions during 1 week (1wME, *n* = 5; representing ongoing open-eye potentiation), 3 weeks (3wME, *n* = 5, representing the end of the open-eye potentiation phase), 5 weeks (5wME, *n = 5*; representing ongoing cross-modal plasticity phase) or 7 weeks (7wME, *n* = 11; representing the end of the cross-modal plasticity phase). All drug-treated ME mice recovered for 7 weeks and received daily intraperitoneal (i.p.) injections of either saline (*n* = 6), 5-HTR_1A_ antagonist (WAY-100635, *n* = 3), 5-HTR_2A_ antagonist (ketanserin, *n* = 3) or 5-HTR_3A_ antagonist (ondansetron, *n* = 3) during the last 3 weeks of the 7 weeks post-ME period (dark gray bar, representing the time window of whisker-driven cross-modal plasticity). The number of animals (n) is represented in the bars

In an animal model of one-eyed vision, due to monocular enucleation (ME) in adult (P120) mice, we could previously establish extensive neuronal reactivation of both the binocular zone (Bz) and the medial monocular zone (Mmz) of the deprived contralateral visual cortex within a 7 week recovery period (7wME) (Fig. 1a, b). This functional recovery not only relied on the expected unimodal potentiation of spared-eye inputs occurring in the Bz during the first 3 weeks post-ME, but also on an ensuing tactile whisker-related reactivation of especially the Mmz from week four onwards [14–16]. This cross-modal effect was most pronounced in the infragranular layers of the Mmz [15]. The level of neuronal activity reached in the Mmz 7 weeks post-ME could be experimentally manipulated by reducing or intensifying whisker inputs, respectively by means of whisker removal or via natural whisker stimulation during exploration of a new, enriched cage environment in complete darkness. These observations confirmed the manifestation of the cross-modal recruitment of the medial monocular cortical territory by the whiskers [15]. The post-ME recovery period is thus characterized by the recruitment of the deprived visual areas by the spared senses in adult mice just as in human patients.

Despite the current understanding of molecular and cellular aspects of visual cortex plasticity [12, 17–22] and the well-described role of neuromodulators in brain plasticity [19, 23–27], only little is known about sensory deprivation-induced alterations in serotonin (5-HT) signaling across sensory areas or, in concreto, about how 5-HT is involved in the different types of cortical plasticity in the context of acquired blindness. A wide range of axonal projections, originating from the serotonergic Raphe Nucleus in the brain stem [28], reach the different sensory cortices, where 5-HT and several of the 14 identified mammalian 5-HT receptor subtypes are involved in the integration of neuronal signals and in the processing of sensory information [29–35].

Of particular interest to the field of cortical plasticity are the serotonergic G-protein coupled receptors 5-HTR_1A_ and 5-HTR_2A_, and ion channel 5-HTR_3A_. These receptors are most abundantly expressed in the mammalian neocortex, predominantly on excitatory, excitatory and inhibitory, and exclusively on inhibitory neurons respectively. In addition, 5-HTR_1A_ and 5-HTR_2A_ have already been implicated in either unimodal or cross-modal plasticity [18, 27, 32, 36–41]. On the one hand, administration of the selective serotonin reuptake inhibitor (SSRI) fluoxetine during a period of visual deprivation via eyelid suture reinstated juvenile-like unimodal ocular dominance plasticity in adulthood. This extraordinary phenomenon was found to be mediated through, amongst others, changes in 5-HTR_1A_ receptor function leading to an experience-dependent shift in the cortical excitation/inhibition balance (E/I) [26, 27, 36, 42, 43]. On the other hand, 2 days of visual deprivation in young rats mediated a 5-HTR_2A/2C_-dependent delivery of AMPAR1 specifically at layer IV-II/III synapses of the primary somatosensory barrel cortex (S1BF), ultimately leading to compensatory plasticity in the form of a sharpened whisker-barrel map and more fine-tuned barrel neuron responses to primary whisker stimulation [32].

Adult cortical plasticity in response to sensory deprivation was further found to involve the activation of dis-inhibitory cortical circuits including the vasoactive intestinal polypeptide (VIP)-positive interneurons [44–51]. VIP cells are a type of 5-HTR_3A_ expressing interneurons, which account for approximately 1/3^rd^ of the entire interneuron population [52]. We discovered before that GABA_A_R_α1_-mediated intracortical inhibition elicits a pivotal role in the different response of the Bz and the Mmz to ME [53]. Since 5-HT and the 5-HT receptors are considered important regulators of the cortical E/I balance [37, 54, 55], they may well exert a major impact on the excitability of the specific brain circuits involved in different types of cortical plasticity.

All these findings indicate that 5-HT may indeed elicit a very important role in the cortical response to sensory loss. We therefore initiated a set of experiments to elucidate if and how exactly 5-HT or one of the three above described 5-HT receptor subtypes, take part in ME-induced plasticity in adult mice. HPLC analysis was performed on whole-tissue homogenates of the visual cortex and S1BF to study the long-term impact of ME on the total 5-HT concentration in these two sensory cortices, known to functionally adapt upon vision loss [15]. Western blotting experiments were conducted to investigate the longitudinal effect of ME on the vesicular monoamine transporter 2 (vMAT2), 5-HTR_1A_ and 5-HTR_3A_ protein expression levels. Different time-points post-ME were chosen to enable distinction between the earlier effects occurring in the Bz, during the unimodal open-eye potentiation phase, and those occurring in the Mmz, during the subsequent cross-modal phase. We pharmacologically antagonized 5-HTR_1A_, 5-HTR_2A_ and 5-HTR_3A_ receptor function, to pinpoint via which of these receptors 5-HT mediates ME-induced cross-modal plasticity in the visual cortex and in S1BF. As before, we relied on in situ hybridization for the neuronal activity reporter gene *zif268* as a high-throughput read-out to differentiate the distinct post-ME plasticity phases (Fig. 1c, d). We demonstrate brain region-specific and time-dependent alterations in pre- and postsynaptic aspects of 5-HT neurotransmission in the adult brain upon ME. A role for 5-HTR_1A_ in unimodal open-eye potentiation was confirmed and we provide evidence for the involvement of 5-HTR_2A_ and 5-HTR_3A_ but not 5-HTR_1A_ in ME-induced cross-modal plasticity. The potential of a defined pharmacological and spatiotemporal control on cross-modal plasticity holds promise towards future refinements of rehabilitation strategies to treat acquired sensory loss.

## Methods

### Animals

In total 54 C57Bl/6 J mice (Janvier Elevage, Le Genest-St-Isle, France) of either sex (32 male/22 female) were used in this study. All mice were housed under standard laboratory conditions with constant room temperature and humidity, an 10/14-h dark/light cycle with food and water available ad libitum. All experiments have been approved by the Ethical Research Committee of KU Leuven and were in strict accordance with the European Communities Council Directive of 22 September 2010 (2010/63/EU) and with the Belgian legislation (KB of 29 May 2013). Every effort was made to minimize animal suffering and to reduce the number of animals. Figure 1 illustrates the experimental manipulations and the number of mice used per condition (Fig. 1b, d). The different phases of cortical plasticity under study have been determined previously based on the impact of either visual stimulation via the spared eye, or somatosensory deprivation/stimulation based on whisker clipping/natural whisker use during the exploration of new toys in complete darkness, on neuronal activity in the visual cortex of adult ME mice [15]. Specifically, 1 week post-ME (1wME) mice are in an ongoing unimodal open-eye potentiation phase. Mice with a 3 week post-ME recovery period (3wME) are at the end of the open-eye potentiation phase, which restores normal visually driven activity levels in an extended binocular zone (Bz). 5 weeks post-ME (5wME) mice are in an ongoing cross-modal phase whereas mice with a 7 week post-ME recovery period (7wME) have undergone maximal cross-modal visual cortex reactivation in which normal activity levels are restored in the monocular zone of the visual cortex, especially medial to the Bz (Mmz), only now relying on whisker inputs. Cortical regions of interest therefore are the visual cortex, Bz and Mmz, and the primary somatosensory barrel field (S1BF).

### Monocular enucleation paradigm and tissue preparation

The removal of the right eye, or monocular enucleation (ME), was performed as described previously [14]. Briefly, adult (P120) mice were anaesthetized by intraperitoneal injection of a mixture of ketamine hydrochloride (75 mg/kg, Dechra Veterinary Products, Eurovet) and medetomidine hydrochloride (1 mg/kg Orion Corporation, Janssen Animal Health). Eye ointment (Tobrex, Alcon) was administered to the left eye to prevent dehydration. The right eye was carefully removed and the orbit was filled with hemostatic cotton wool (Qualiphar, Bornem, Belgium) in case of bleeding. Analgesics were injected subcutaneously (Metacam, 2 mg/kg, 0.1 mL) and atipamezol hydrochloride was administered to reverse anaesthesia (1 mg/kg i.p., Orion Corporation, Elanco Animal Health). Following ME, the mice were housed in their home cages under standard laboratory conditions for a 1 to 7 week recovery period. Control mice are age-matched (AMC) to the 7 week ME mice, the time point of maximal recovery of neuronal activity [56]. At the end of the ME period, the mice were sacrificed by cervical dislocation. For HPLC-based determination of the total 5-HT concentration in visual and somatosensory tissue samples, the brains were rapidly extracted and immediately frozen in 2-methylbutane (Merck, Overijse, Belgium) at a temperature of − 40 °C. All brains were stored at − 80 °C until sectioning. A tissue blotting device (Model PA 002 Mouse Brain Blocker, 1 mm, David KOPF instruments, California) was used to prepare approximately 1 mm-thick coronal slices to subsequently isolate the whole visual cortex and the primary somatosensory cortex (S1BF) with sterile scalpels. Separate analysis of medial monocular and binocular cortex was technically impossible with this method. For Western blotting experiments, 100 μm-thick coronal cryosections were collected on baked glass slides and stored at − 80 °C. Specifically for radioactive in situ hybridization (ISH) experiments, the mice were placed overnight in their home cages in a dark room to reduce *zif268*-expression to basal levels. The following day, the mice were placed in a high-lit environment to upregulate sensory driven *zif268*-mRNA expression. After 45 min, at peak *zif268*-mRNA expression levels, the mice were sacrificed by cervical dislocation. The brains were rapidly removed, immersed in 2-methylbutane at a temperature of − 40 °C (Merck, Overijse, Belgium) and stored at − 80 °C until sectioning. Serial sections with a thickness of 25 μm were prepared on a cryostat (HM 500 OM, Microm, Thermo Scientific, Walldorf, Germany), mounted on 0.1% poly-L-Lysine-coated (Sigma-Aldrich) slides, and stored at − 20 °C until further processing.

### Quantification of the total serotonin content in mouse brain homogenates

**Fig. 2:**
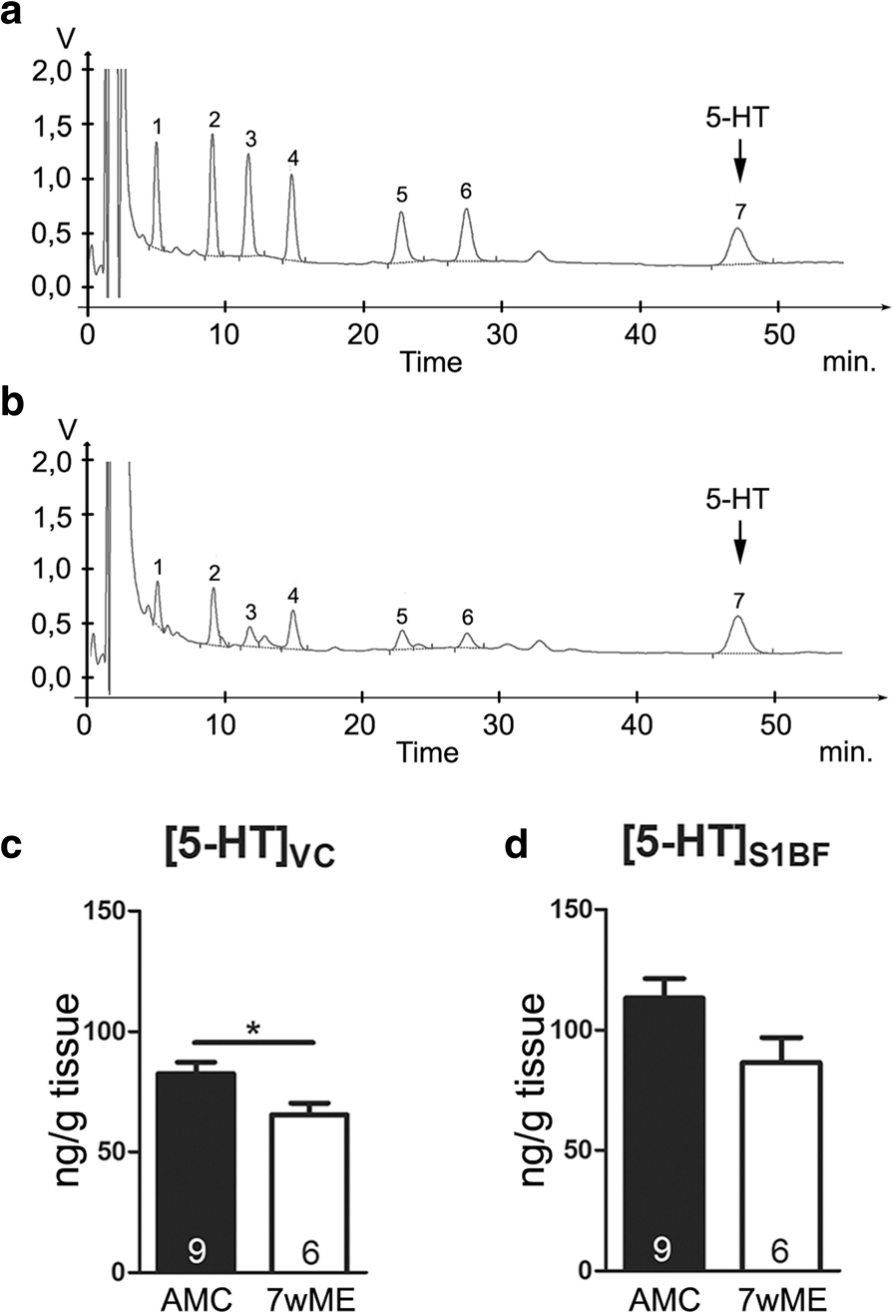
ME-induced decrease in total 5-HT concentration in the adult visual cortex. **a, b** HPLC chromatogram of respectively the standard and a cortical sample. The peaks were numbered from 1 to 7 and represent noradrenaline; 3,4-dihydroxybenzylamine; 3,4-dihydroxyphenylacetic acid; dopamine; 5-hydroxy-indoleacetic acid; homovanillic acid and serotonin (5-HT). **c** In the visual cortex (VC) of 7wME mice (*n* = 6), a significantly decreased total 5-HT concentration was observed compared to AMC mice (*n* = 9, *p* = 0.018, Mann-Whitney test). **d** The primary somatosensory barrel field (S1BF) did not show a significant difference (*p* = 0.114, Mann-Whitney test). These results indicate an ME-induced effect on 5-HT signaling in sensory deprived cortex. The number of animals (n) is represented in the bars. *P < 0.05

The total serotonin (5-HT) content in the visual and barrel cortex (HPLC total *n* = 15, AMC: *n* = 9; 7wME: *n* = 6) was measured based on previously reported methods [57–59]. In summary, after weighing cortical tissue, 190 μL of an antioxidant solution (0.1 M acetic acid, 3.3 mM L-cysteine, 0.27 mM Na_2_EDTA and 0.0125 mM ascorbic acid) and 10 μL of an internal standard solution (3,4-dihydroxybenzylamine solution 1 μg/mL in antioxidant) were added to the tissue. After homogenization, the samples were centrifuged (20 min, 9500 g, 4 °C). The supernatant was diluted 5-fold in 0.5 M acetic acid and 20 μL was injected automatically on a reversed phase liquid chromatography system (autosampler ASI-100 and HPLC pump P680 A HPG/2, Dionex, Amsterdam, The Netherlands) with electrochemical detection (potential = + 700 mV) (Amperometric Detector LC-4C, BAS, Indiana, USA). With this liquid chromatography method, we are able to measure within one run the monoamines noradrenaline, dopamine and 5-HT, some of their major metabolites (such as 3,4-dihydroxyphenylacetic acid; 5-hydroxy-indoleacetic acid; homovanillic acid) as well as the internal standard 3,4-dihydroxybenzylamine (Fig. 2). The separation between the different compounds was achieved using a narrowbore C18 column (Alltech®, Alltima™, 5 μm, 150 × 2.1 mm, Grace, Deerfield, IL, USA). The mobile phase buffer contained 0.1 M sodium acetate, 20 mM citric acid, 1 mM sodium octane sulfonic acid, 1 mM dibutylamine and 0.1 mM Na_2_EDTA adjusted to pH 3.7 (mobile phase composition: 97 buffer / 3 methanol (*v*/v)). For this study, we specifically quantified the 5-HT content of the different samples. Tissue concentration was expressed as ng 5-HT /g wet tissue (ng/g).

### Western analysis

**Fig. 3:**
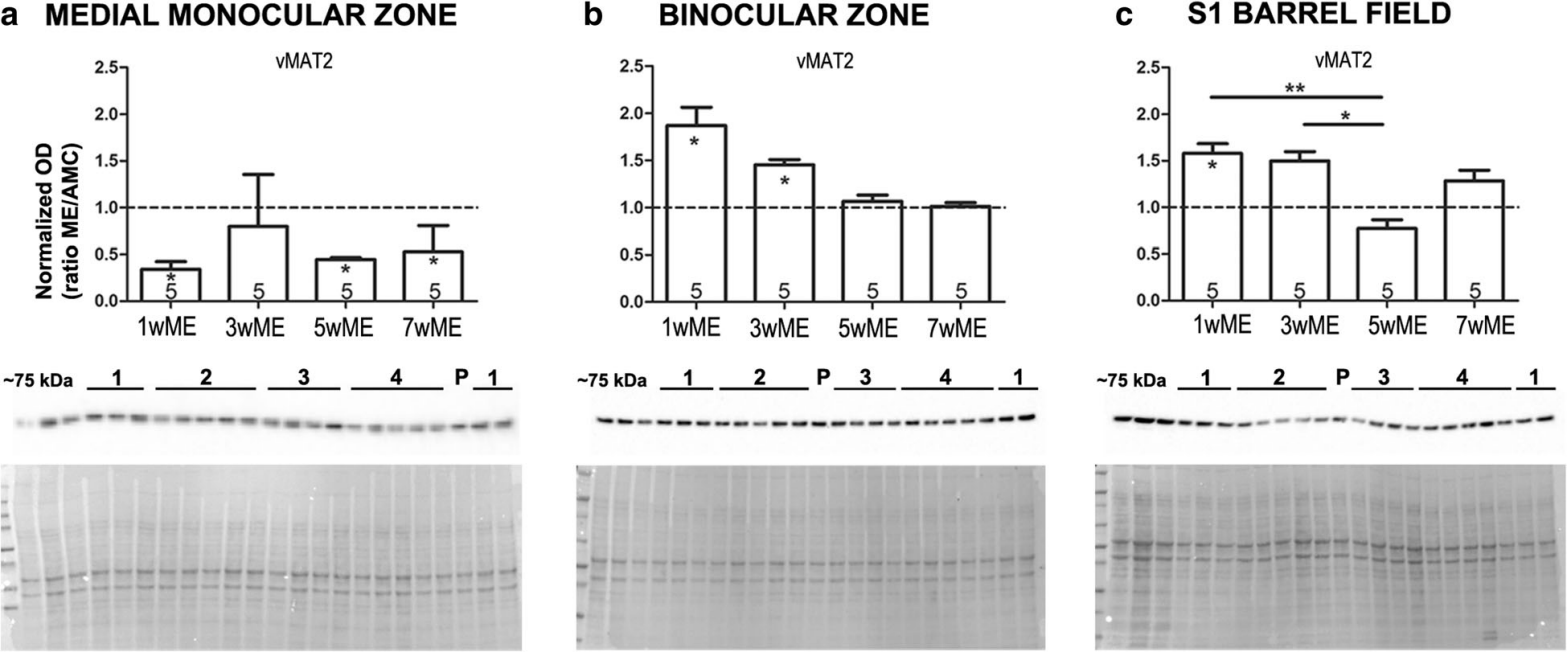
Time-dependent effect of ME on cortical area-specific vMAT2 expression levels. Western blot OD values are illustrated as the ratio of the expression levels of each ME group relative to the expression of the AMC group to judge the effect of post-ME recovery time. Below each graph, representative Western blot bands are depicted, as well as the expected molecular weight (~ 75 kDa) and an example total protein stain. The different conditions are indicated with numbers above the respective bands: 1 = 3wME, 2 = 5wME, 3 = AMC, 4 = 7wME and *P* = region-specific tissue sample pool. **a** At 1 week post-ME (1wME), the expression of vesicular monoamine transporter 2 (vMAT2) in the medial monocular zone (Mmz) was significantly reduced compared to the AMC mice (dashed line) and the vMAT2 expression levels remain low, even after 7 weeks. **b** In the binocular zone (Bz), a significant ME-effect was detected in 1wME and 3wME mice, showing increased vMAT2 expression levels immediately after ME. **c** In the primary somatosensory barrel field (S1BF), vMAT2 expression was increased in 1wME mice compared to AMCs, and was significantly higher in 1wME and 3wME mice compared to 5wME mice. The number of animals (n) is represented in the bars. Significant pair-wise differences between ME mice and the AMC group are indicated with a ‘*’ within the corresponding bar. Differences between ME conditions due to post-ME recovery time are indicated as a ‘*’ above the respective ME conditions. *P < 0.05, **P < 0.01

**Fig. 4:**
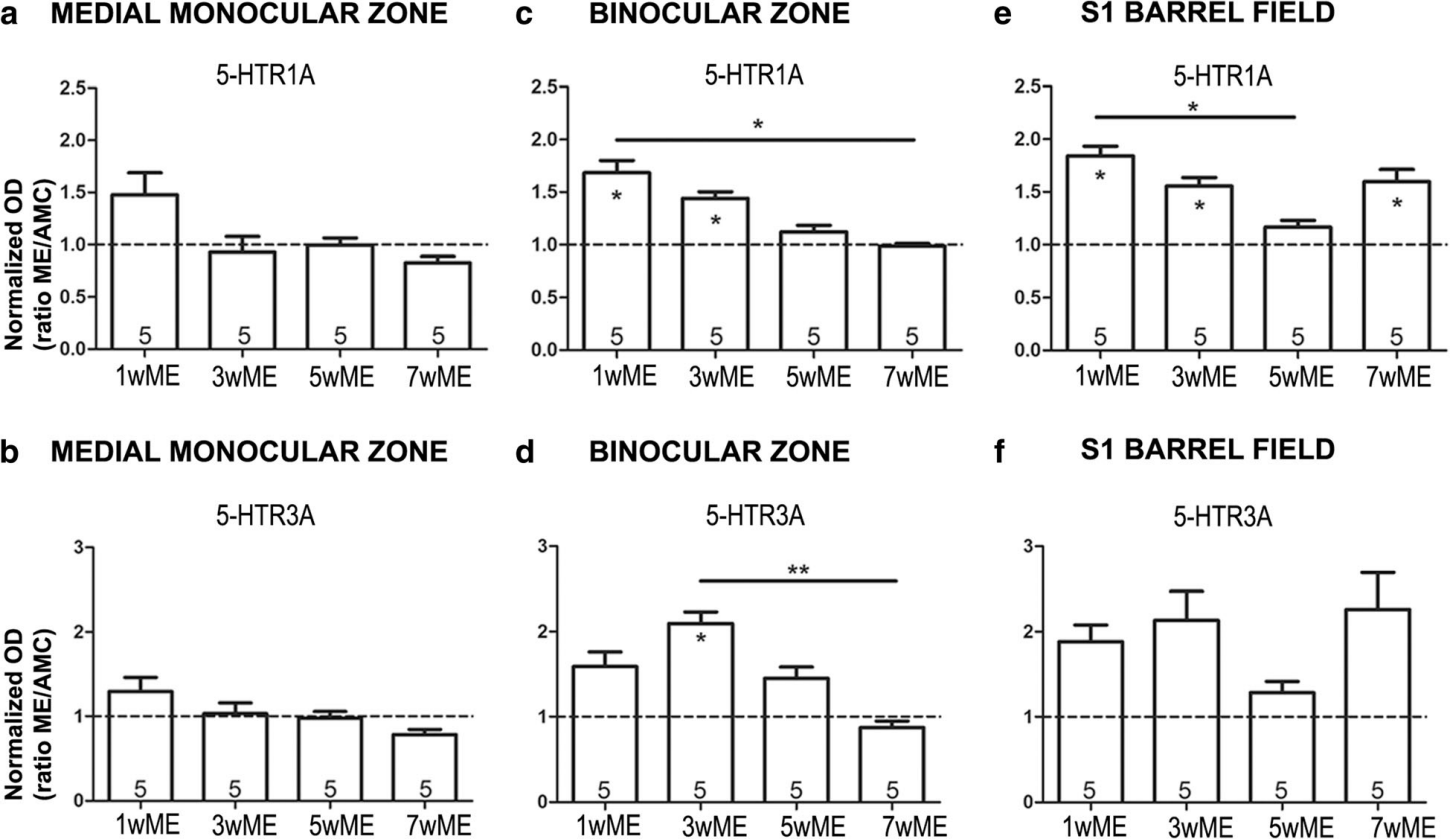
Time-dependent effect of ME on cortical area-specific 5-HTR_1A_ and 5-HTR_3A_ expression levels. **a, b** Analysis of the Western blot OD values, illustrated as the ratio of the expression level of each ME group relative to the expression of the age-matched control group (AMC, dashed line) to judge the effect of post-lesion recovery time, revealed no significant impact of ME on 5-HTR_1A_ and 5-HTR_3A_ receptor expression, and also no time-dependent modulations in the medial monocular zone (Mmz) of the visual cortex of ME mice. **c** In the binocular zone (Bz), post-ME 5-HTR_1A_ expression levels were increased in 1wME and 3wME mice and gradually decreased over time, reaching AMC levels in 5wME and 7wME mice. **d** The 5-HTR_3A_ receptor levels in the Bz, were significantly higher in 3wME mice compared to AMCs and decreased gradually over time to reach AMC levels at 5 and 7 weeks post-ME. **e** In the primary somatosensory barrel field (S1BF), the 5-HTR_1A_ expression was increased in 1wME and 3wME mice, decreased over time to reach its lowest expression levels at 5 weeks post-ME (5wME) and again increased at 7 weeks post-ME. **f** Although similar ME-induced and time-dependent modulations in 5-HTR_3A_ expression were observed in S1BF, they did not reach statistical difference, possibly due to larger inter-individual variation. The number of animals (n) is represented in the bars. Significant pair-wise differences between ME mice and the AMC group are indicated with a ‘*’ within the corresponding bar. Differences between ME conditions due to post-ME recovery time are indicated as a ‘*’ above the respective ME conditions. *P < 0.05, **P < 0.01

Western blotting (WB) was used to investigate the changes in relative expression of pre- and postsynaptic proteins involved in serotonergic neurotransmission. Time-course samples were prepared to separately examine the protein expression in the Mmz, Bz and S1BF over a 7-week period. The experimental conditions included: 1, 3, 5, 7 weeks post-ME. For each of the experimental conditions, as well as for the control mice age-matched to the 7wME mice (AMC), at least 4 mice were included (Figs. 3 and 4, WB total *n* = 24, AMC: *n* = 4; 1wME: *n* = 5; 3wME: *n* = 5; 5wME: *n* = 5; 7wME: *n* = 5). All ME samples were analyzed relative to the AMC samples (absolute OD-values measured in the Mmz, Bz or S1BF of the AMC as mean ± SEM for vMAT2 (Mmz: 2.824 ± 0.483; Bz: 0.691 ± 0.044; S1BF: 0.953 ± 0.130), 5-HTR_1A_ (Mmz: 0.855 ± 0.126; Bz: 0.667 ± 0.076; S1BF: 0.700 ± 0.073) and 5-HTR_3A_ (Mmz: 0.955 ± 0.087; Bz: 0.854 ± 0.138; S1BF: 0.764 ± 0.273)). We chose to analyze vMAT2 protein expression levels since they provide information on the presynaptic loading of vesicles with 5-HT and because vMAT2 expression levels positively correlate with the total 5-HT concentration present in a given brain region of interest [60–62].

### Protein extraction from tissue slices

Based on the mouse brain atlas of Paxinos and Franklin (2013) [63], the primary somatosensory barrel field (S1BF: 0.5-(− 2); 3–4.5; 1, relative to Bregma in A-P; M-L; D), the medial monocular zone (Mmz, comprising monocular V1 and V2M: − 2.7-(− 4.7); 1–2.5; 1, relative to Bregma and the binocular zone (Bz, comprising binocular V2L and V1: − 2.7-(− 4.7); 2.5–4; 1, relative to Bregma) were microscopically excised and collected separately from 100 μm-thick coronal cryosections **(**Fig. 1c**)**. Tissue was collected in a mix of 4 μL of complete protease inhibitor cocktail (Roche Diagnostics, GmbH) and 100 μL ice-cold lysis buffer (2% SDS, 65 mM Tris-HCl in MQ) optimized for the enrichment of membrane (−associated) proteins [64, 65]. Proteins were extracted from the tissue by mechanical homogenization using drill-driven, sterile disposable pestles (Argos Technologies), sonication (5 × 10 s), incubation at 70 °C (5 min) and centrifugation (15 min, 13000 rpm, 4 °C). The supernatant was collected and the total protein concentration was determined using the Qubit fluorometer (Invitrogen). Samples were stored at − 80 °C.

### Immunoblotting

To obtain the optimal primary antibody working concentration, a protein dilution series ranging from 5 to 30 μg was analyzed. A concentration within the linear range of the detection system that resulted in a good signal to noise ratio was chosen for monocular, binocular and somatosensory samples separately. For vMAT2, 5-HTR_1A_ and 5-HTR_3A_ analysis, this resulted in 15 μg for samples of all three regions. 5-HTR_2A_ was excluded from the analyses due to the lack of a specific antibody. Reference sample (pool) consisting of a mixture of equal amounts of each prepared tissue sample was run for monocular, binocular and somatosensory cortex, with the same optimal amount of protein on each gel to gauge blot-to-blot variability. After the addition of 5 μl reducing agent (10×, Invitrogen, Paisley, United Kingdom) and 2 μl LDS sample buffer (4×, Invitrogen), the samples were denatured (10 min, 70 °C). The protein samples were separated on 4–12% Bis-Tris Midi-gels in the XCell4 SureLock Midi-Cell (Invitrogen). The Spectra™ Multicolor Broad range protein ladder (ThermoScientific) was used as molecular weight standard. Subsequently, the samples were transferred to a polyvinylidene fluoride (PVDF) membrane. After 1–2 h incubation in a 5% ECL blocking agent (GE Healthcare, Buckinghamshire, UK) in Tris-saline (0.01 M Tris, 0.9% NaCl, 0.1% TX-100, pH 7.6), the membrane was incubated overnight with a primary antibody rabbit anti-vMAT2 (1:1000, R&D Systems, Novus Biologicals), with rabbit anti-5-HTR_1A_ (1:200, Alomone Labs), with rabbit anti-5-HTR_3A_ (1:200, Alomone Labs). The next day, the blots were successively washed in Tris-saline (4 × 5 min), 30 min incubated with HRP-conjugated secondary antibody (goat anti-rabbit IgG, 1:50.000, Dako, Glostrup, Denmark), rinsed in Tris-saline (5 × 5 min) and Tris-stock (1 × 5 min) (0.05 M Tris, pH 7.6). The immunoreactive bands were visualized using a chemiluminescent reaction (1 × 5 min, Super Signal West Dura, ThermoScientific, Pierce) combined with the BIO-RAD ChemiDoc™ MP Imaging System. In order to correct for intra- and inter-gel variability and to normalize the concentration of the specific detected proteins to the total amount of protein present, we performed a total protein stain (TPS) with Swift Membrane Stain (G-Biosciences) according to manufacturer’s instructions. Immediately after the staining, blots were scanned with the BIO-RAD ChemiDoc™ MP Imaging System.

### Semi-quantitative Western analysis

The immunostained protein bands were semi-quantitatively evaluated by densitometry (Image Lab™ software) separately for monocular, binocular and somatosensory cortex samples **(**Figs. 3 and 4**)**. First, to account for intra-gel and inter-gel variability including loading differences or incomplete transfer onto the membrane, a TPS was employed rather than the use of a single reference protein [53, 66, 67]. For each protein of interest, the optical density value per mouse was normalized to its corresponding TPS. Also, to compare samples between different gels, normalized data were expressed relative to the region-specific reference sample (pool).

### Pharmacology

ME mice (pharmacology total *n* = 15, saline: *n* = 6; WAY-100635: *n* = 3; ketanserin: *n* = 3; ondansetron: *n* = 3) received daily injections (i.p.), at 2 pm exactly, of a specific 5-HT receptor antagonist at a dose based on prior literature, and specifically during the 3-week period of cross-modal plasticity from week 5 to 7 post-ME (Fig. 1d). Systemic drug delivery was chosen because a long-term treatment with a locally implanted slow release system (e.g. Alzet minipump) would lead to cortical tissue damage and scar formation, potentially influencing the local neuromodulator levels [68]. Drugs used were the 5-HTR_1A_ antagonist WAY-100635 maleate (Abcam Ab120550, 1 mg/kg, 0.2 mL i.p.) [37, 36, 69, 70], the 5-HTR_2A_ antagonist ketanserin tartrate (R&D Systems, Tocris Bioscience, 5 mg/kg, 0.2 mL i.p.) [71–74] and the 5-HTR_3A_ antagonist ondansetron hydrochloride (R&D Systems, Tocris Bioscience, 5 mg/kg, 0.2 mL i.p.) [75]. Control animals were housed under the same standard conditions and received saline injections (0.9%, i.p.) following the same injection regimen. All animals were sacrificed by cervical dislocation. Brains were rapidly removed and stored as described above.

### In situ hybridization for *zif268*-mRNA

High-throughput radioactive ISH experiments were performed on series of coronal brain sections between Bregma levels − 1.5 mm and − 5 mm and changes in the mRNA expression level of the immediate early gene (IEG) *zif268*, a proven excellent activity reporter gene in the mammalian brain, were quantified (mouse: [15, 16, 51, 53, 56, 76–78], cat: [79–82]. As such, the spatial extent and the exact anatomical location of experience-induced, predominantly excitatory [6, 83–87], neuronal activity changes were analyzed and compared throughout all cortical layers of the visual and somatosensory neocortex. This high-throughput approach allows the molecular visualization of cortical reactivation patterns in response to ME. ISH for *zif268*-mRNA was performed with a mouse-specific synthetic oligonucleotide probe (Eurogentec, Seraing, Belgium) with sequence 5′-ccgttgctcagcagcatcatctcctccagtttggggtagttgtcc-3′. As described previously [51, 53, 77, 88], each probe was 3′-end labeled with [^33^P] dATP using terminal deoxynucleotidyl transferase (Invitrogen, Carlsbad, CA). Unincorporated nucleotides were separated from the labeled probe by means of miniQuick Spin Oligo Columns (Roche Diagnostics, Vilvoorde, Belgium). The cryostat sections were fixed, dehydrated and delipidated. The radioactively labeled probes were added to a hybridization cocktail (50% (*v*/v) formamide, 4× standard SSC buffer, 1× Denhardt’s solution, 10% (*w*/*v*) dextran sulfate, 100 μg/ml herring sperm DNA, 250 μg/ml tRNA, 60 mM dithiothreitol, 1% (w/v) N-lauroyl sarcosine, and 20 mM NaHPO_4_, pH 7.4) and applied to the cryostat sections (10^6^ cpm/section). After an overnight incubation at 37 °C in a humid chamber, the sections were rinsed in 1× standard SSC buffer at 42 °C, dehydrated, air-dried and exposed to an autoradiographic film (Biomax MR; Kodak, Rochester, NY). After 7 days, the films were developed in EMS replacement for Kodak developer D-19 (Electron Microscopy Sciences, Hatfield) and fixed in Rapid fixer (Ilford Hypam; Kodak). Autoradiographic images of adjacent sections per examined cortical area per mouse were scanned at 1200 dpi (CanoScan LIDE 600F; Canon, Tokyo, Japan), and all images were similarly adjusted for brightness and contrast in Adobe Photoshop Elements 2018 (version 16.0, × 64, Adobe Systems Incorporated).

### Histology and localization of visual and somatosensory areal boundaries with Nissl patterns

**Fig. 5:**
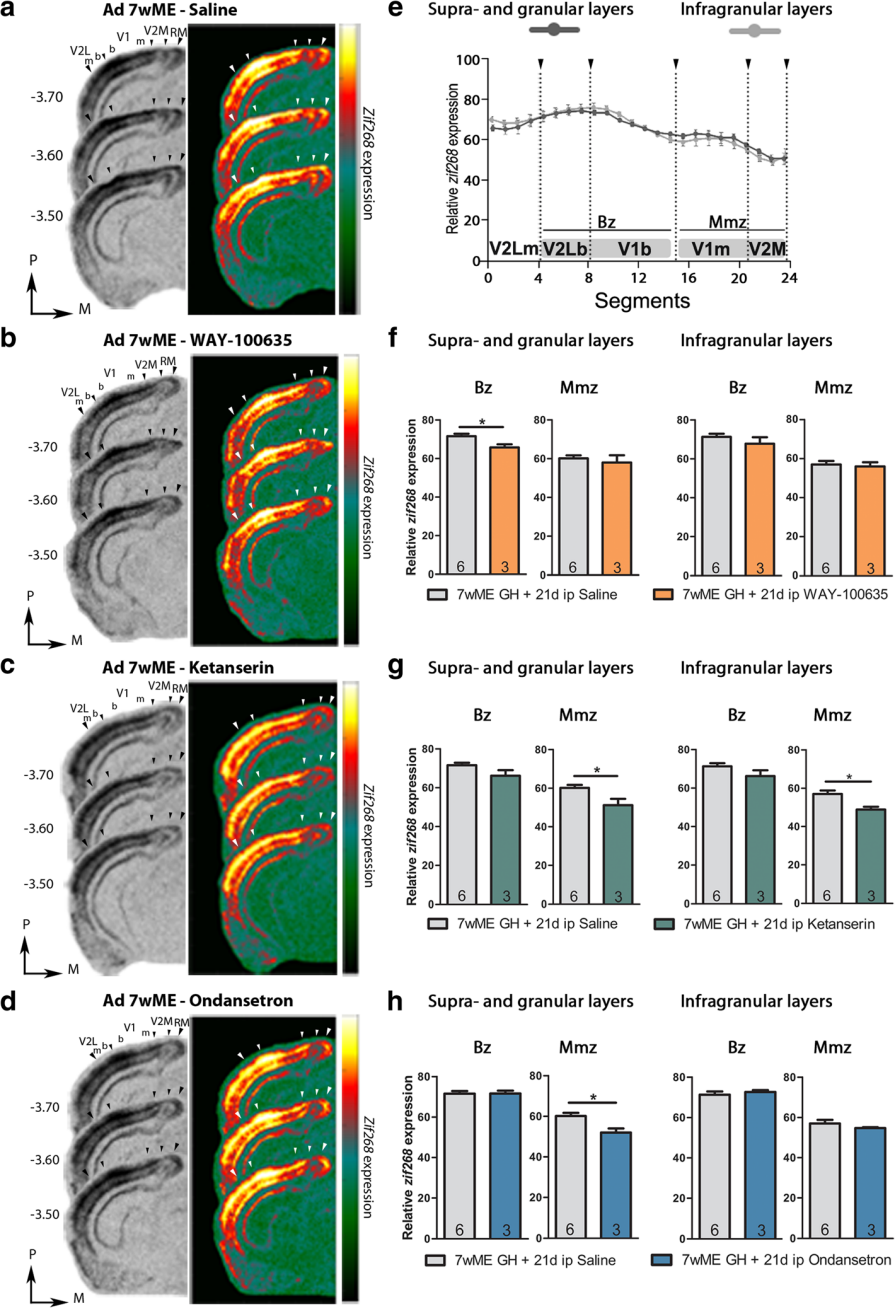
5-HTR antagonist treatment from week 5–7 post-ME affects neuronal activity levels in Mmz and Bz. **a-d** Images of 3 adjacent sections (− 3.5-(− 3.7); 1–4; 1, relative to Bregma) from adult 7wME mice, drug or sham-treated during the last 3 weeks of the 7wME recovery period: (**a**) saline, (**b**) 5-HTR_1A_-antagonist WAY-100635, (**c**) 5-HTR_2A_-antagonist ketanserin, (**d**) 5-HTR_3A_-antagonist ondansetron. Corresponding pseudocolor representations are displayed next to their respective ISH sections. **e** Line graphs illustrating relative *zif268*-mRNA expression levels, measured as the average OD-value/segment, for saline-injected 7wME mice. For supra- and granular layers II/III-IV (e, left panel, dark gray line) and for infragranular layers V-VI (e, right panel, light gray line) the expression levels are displayed along the 5 predefined visual subdivisions (black arrowheads are in accordance with the small arrowheads in a-d, including the subdivision between monocular zones m and binocular zones b in V2L and V1). Error bars represent the SEM of the mean OD value in each segment. Relative *zif268*-mRNA expression levels are shown as OD-values averaged over the binocular zone Bz and the medial monocular zone Mmz for supra- and granular layers (**f-h**, left panel) and infragranular layers (**f-h**, right panel). Bz comprises V2Lb-V1b (dark gray marking), Mmz includes V1 m-V2M (light gray marking) as illustrated in **e**. Comparison of the OD-values of saline-injected (light gray bars) and WAY-100635-injected 7wME mice (orange bars) indicates decreased reactivation levels in the upper layers of the Bz (**f**, left panel). Comparison of the OD-values of saline-injected (light-gray bars) and ketanserin-treated 7wME mice (green bars) indicates decreased reactivation levels across all layers of the Mmz (**g**). Comparison of the OD-values of saline-injected (light-gray bars) and ondansetron-treated 7wME mice (blue bars) indicates decreased reactivation in the upper layers of the Mmz (**h**, left panel). The number of animals (n) is shown in the bars. *P < 0.05

**Fig. 6:**
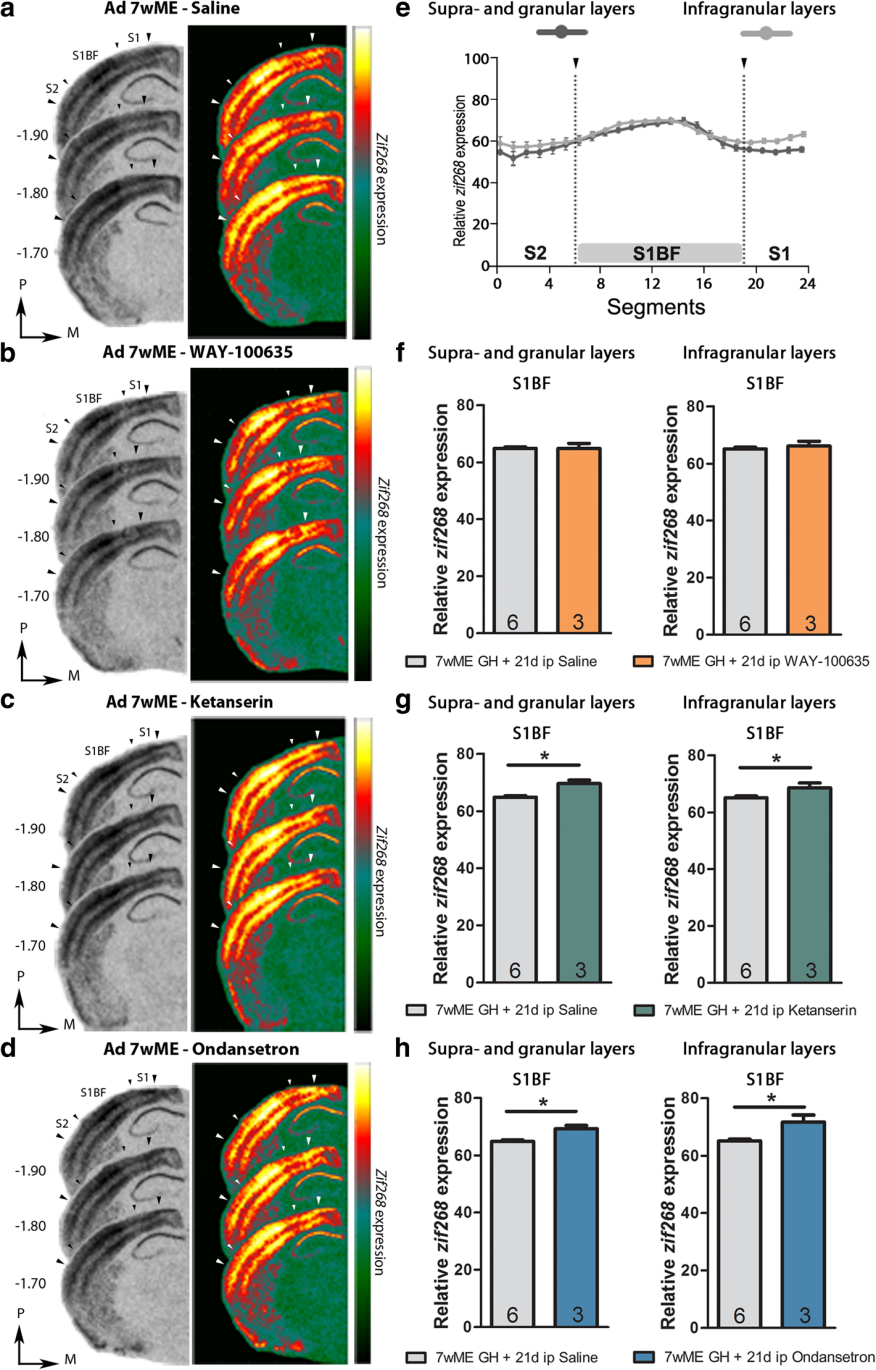
5-HTR antagonist treatment from week 5–7 post-ME affects neuronal activity levels in S1BF. **a-d** Images of 3 adjacent sections (− 1.7-(− 1.9); 2.5–4; 1, relative to Bregma) from adult 7wME mice, injected during the last 3 weeks of the 7wME recovery period with (**a**) saline, (**b**) 5-HTR_1A_ antagonist WAY-100635, (**c**) 5-HTR_2A_ antagonist ketanserin, (**d**) 5-HTR_3A_ antagonist ondansetron. Corresponding pseudocolor representations are displayed next to their respective ISH sections. **e** Line graphs illustrating the relative *zif268*-mRNA expression level, measured as the average OD-value/segment, for saline-injected 7wME mice. For supra- and granular layers II/III-IV (e, left panel, dark gray line) and for infragranular layers V-VI (e, right panel, light gray line) the expression levels are displayed along the 3 predefined somatosensory subdivisions (black arrowheads are in accordance with the small arrowheads in a-d). Error bars represent the SEM of the mean OD value in each segment. Relative *zif268*-mRNA expression levels are shown as OD-values averaged over the primary somatosensory barrel cortex (S1BF) for supra- and granular layers (**f-h**, left panel) and infragranular layers (**f-h**, right panel). Comparison of the OD-values of saline-injected (light gray bars) and WAY-100635-injected 7wME mice (orange bars) indicates no post-ME changes in neuronal activity in S1BF due to long-term i.p. injection of 5-HTR_1A_ antagonist (**f**). Comparison of the OD-values of saline-injected (light-gray bars) and ketanserin-treated 7wME mice (green bars) indicates increased neuronal activity across all layers of the S1BF (**g**). Comparison of the OD-values of saline-injected (light-gray bars) and ondansetron-treated 7wME mice (blue bars) indicates increased neuronal activity across all layers of the S1BF (**h**). The number of animals (n) is represented in the bars. *P < 0.05

Upon ISH, the cryostat sections were Nissl-counterstained (1% cresyl violet; Fluka, Sigma-Aldrich) according to standard procedures to visualize cortical boundaries between different visual and somatosensory areas and to aid the interpretation of the *zif268*-activity patterns (Fig. 1c). Images of the stained coronal sections were obtained at 5× (NA: 0.16) with a light microscope (Zeiss Axio Imager Z1) equipped with an AxioCam MRm camera (1388 × 1040 pixels) using the software program Zen (Zen Pro 2012, Carl Zeiss, Benelux). Comparisons were made with the stereotaxic mouse brain atlas [63] to delineate visual and somatosensory cortical borders as described previously [53, 56]. In all figures illustrating visual or somatosensory cortex, large arrowheads indicate the total extent of the cortex, whereas small arrowheads indicate the interareal borders. In the visual cortex, five subregions can be distinguished from lateral to medial (Figs. 1c, 5): the lateral extrastriate cortex (V2L), which is subdivided into a monocular (V2Lm, segments 1–4) and binocular region (V2Lb, segments 4–8), the primary visual cortex (V1) which is subdivided further into a binocular (V1b, segments 8–15) and monocular region (V1 m, segments 15–21), and the medial extrastriate cortex (V2M, segments 21–24) [51, 53, 56, 89]. For the *zif268* analysis, we focused specifically on the Bz (V2Lb-V1b) and the Mmz (V1 m-V2M) as these regions undergo open-eye potentiation and cross-modal plasticity respectively. In the somatosensory cortex, we distinguished the primary somatosensory barrel field (S1BF) from the more lateral secondary somatosensory cortex (S2) and the more medial primary somatosensory cortex (S1), as the primary receiver of whisker inputs (Fig. 6) [63].

### Quantitative analysis of ISH results

To quantify the optical density (OD; mean gray value per pixel) of the ISH autoradiograms, a custom-made Matlab (Matlab R2017a; Mathworks) script was used as described previously [51, 53]. We analyzed at least three mice per condition. Per mouse, three ISH sections with an inter-distance of 100 μm were investigated (for visual cortex: − 3.5-(− 3.7); 1–4; 1, relative to Bregma; for somatosensory cortex: − 1.7-(− 1.9); 2.5–4; 1, relative to Bregma). The region of interest in the left hemisphere was demarcated by determining the top edge of the cortex, the boundary between the supra-and granular layers (II-III and IV) and the infragranular layers (V and VI), and the border between the infragranular layers and the corpus callosum. The region of interest was then divided equally into 24 segments from lateral to medial to create two lattices of 24 quadrangles, corresponding to the upper (II-IV) and lower (V-VI) layers. To compensate for possible variation in brain size and morphology, the lattices were translated on each autoradiogram over the cortical curvature, fixing the border of a specific segment to an areal border (border segment 20/21 is the area border V1 m/V2M). For each segment created this way, the relative OD was calculated as the mean gray value of all pixels contained within a particular quadrangle and was normalized to the mean gray value of a square measured in the corpus callosum (a defined region with no *zif268*-mRNA expression above background) in order to compare autoradiograms across experiments. Relative neuronal activity was expressed in percentages based on the following formula: 1 – (cortical *zif268*/background) × 100. Results are presented as profiles of neuronal activity per brain area and are illustrated in subregion-specific bar graphs. Pseudo-color maps were generated through a second custom-made Matlab script (Matlab R2017a; Mathworks, Natick, MA) and represent a false color coding of the gray values ranging from black (0) to white (225); high gray values are represented in white/yellow on the false color scale bar (0–50), medium high gray values are represented in red (50–100) or blue (100–150) and low gray values are represented in green/black (150–255).

### Statistics

All HPLC results, Western blotting data and relative OD-values in ISH-sections were presented as mean ± SEM. Normal distribution and in parallel, equal variance between groups was tested. A non-parametric test (Mann-Whitney) was applied for pairwise comparison. A Kruskal-Wallis test followed by a Dunn’s multiple comparisons post-hoc test was used to determine the recovery time-dependent modulations of protein expression levels upon ME. For all tests, a probability level (α level was set to 0.05) of < 0.05 was accepted as statistically significant (*p < 0.05, **p < 0.01, ***p < 0.001). Statistical analyses were performed using GraphPad Prism 5.01 (GraphPad Software, Inc).

## Results

### Reduced visual cortex 5-HT concentration in response to monocular enucleation

To investigate if 5-HT is involved in adult ME-induced cross-modal plasticity and to better understand its role therein, we first examined whether the total 5-HT concentration was affected in the two cortices that functionally adapt upon ME. The total 5-HT concentration was analyzed with High-Performance Liquid Chromatography (HPLC) on whole tissue homogenates of the visual cortex and S1BF 7 weeks after performing ME in adult mice (7wME) (Fig. 2a, b). At this 7wME endpoint, when the cross-modal recruitment of the initially deprived visual cortex is completed [15], we observed a decreased 5-HT concentration in the visual cortex (mean ± SEM, AMC: 82.54 ± 4.77; 7wME: 65.50 ± 4.77, Mann-Whitney test, *p* = 0.018, Fig. 2c)**.** In S1BF, the cortical area in which compensatory plasticity is triggered by vision loss, no significant difference was reached **(**AMC: 113.40 ± 8.06; 7wME: 86.41 ± 10.49; Mann-Whitney test, *p* = 0.114, Fig. 2d). These results suggest brain region-specific modulations of total 5-HT content in response to visual deprivation.

### ME induces pre- and postsynaptic changes in proteins related to 5-HT signaling

Parallel to the ME-induced decrease in total 5-HT concentration in the visual cortex, the impact of partial vision loss might manifest at the level of 5-HT loading into readily releasable vesicles or at the level of 5-HT receptors. To investigate possible brain region-specific expression changes in these pre- and postsynaptic proteins involved in 5-HT signaling, we performed Western blotting experiments on time-course samples from the visual Mmz and Bz separately and from S1BF. Post-ME recovery periods of respectively 1 week (1wME: ongoing open-eye potentiation phase), 3 weeks (3wME: end of the open-eye potentiation phase) and 5 weeks (5wME: ongoing cross-modal phase) were chosen as intermediate time points towards the time point of maximal visual cortex reactivation (7wME) for the evaluation of possible post-ME recovery time-dependent modulations in protein expression in addition to endpoint evaluation (Fig. 1) [15].

We chose to analyze vMAT2 protein expression levels since they provide information on the presynaptic loading of vesicles with 5-HT and because vMAT2 expression levels positively correlate with the total 5-HT concentration present in a given brain region of interest [60–62]. vMAT2 expression levels were significantly decreased in the Mmz in 1wME, 5wME and 7wME mice (represented as * within the bar, bar graphs in Figs. 3 and 4) compared to AMC (represented as a dashed line in Figs. 3 and 4, mean ± SEM, 1wME: 0.34 ± 0.08, *p* = 0.0159; 3wME: 0.80 ± 0.56, *p* = 0.4127; 5wME: 0.45 ± 0.02, *p* = 0.0159; 7wME: 0.53 ± 0.28, *p* = 0.0317, Mann-Whitney test). No statistically significant modulations occurred as a function of post-ME recovery time (Kruskal-Wallis test: *p* = 0.0159, with no statistical difference in the Dunn’s post-hoc test for pairwise comparison) (Fig. 3a). Western analysis of the vMAT2 expression in the Bz indicated increased vMAT2 expression in 1wME and 3wME mice compared to AMC mice (mean ± SEM, 1wME: 1.87 ± 0.19, *p* = 0.0317; 3wME: 1.45 ± 0.06, *p* = 0.0159; 5wME: 1.07 ± 0.07, *p* = 0.4127; 7wME: 1.01 ± 0.04, *p* = 0.7302, Mann-Whitney test). As for the Mmz, also for the Bz no significant recovery time-dependent changes were observed (Kruskal-Wallis test: *p* = 0.0095, with no statistical differences in the Dunn’s post-hoc test for pairwise comparison). In this case, however, a trend of decreasing vMAT2 expression was observed over the 1w to 7w post-ME time course (Fig. 3b). In S1BF, vMAT2 expression was significantly increased in 1wME mice compared to AMC (mean ± SEM, 1wME: 1.58 ± 0.11, *p* = 0.0317; 3wME: 1.50 ± 0.10, *p* = 0.0635; 5wME: 0.77 ± 0.09, *p* = 0.1905; 7wME: 1.29 ± 0.11, *p* = 0.4127, Mann-Whitney test, Fig. 3c). Furthermore, in 1wME and 3wME mice, the vMAT2 expression levels were higher compared to 5wME mice (Kruskal-Wallis test: *p* = 0.0081). These results point towards a return to baseline vMAT2 expression levels specifically at the time when the cross-modal whisker take-over of the deprived visual cortex starts to occur [15].

To define the impact of ME on the serotonergic modulation of cortical inhibition through specific 5-HT receptors, we performed Western blotting experiments for 5-HTR_1A_ and 5-HTR_3A_. Besides their role in different types of visual cortex plasticity, reasons supporting these receptor choices, are the fact that the 5-HTR_1A_ receptor is expressed both on pre-and postsynaptic neurons, respectively acting as an autoreceptor and a Gi-coupled GPCR mediating inhibitory neurotransmission upon ligand binding [90], while 5-HTR_3A_ is a Na^+/^K^+/^Ca^2+^ permeable ion channel exclusively expressed on the 5-HTR_3A_ expressing inhibitory interneurons [91]. We did not observe any ME-induced or recovery time-dependent changes in the expression of 5-HTR_1A_ or 5-HTR_3A_ in the Mmz **(**mean ± SEM, 5-HTR_1A_, 1wME: 1.48 ± 0.21, *p* = 0.2857; 3wME: 0.93 ± 0.15, *p* = 0.7302; 5wME: 1.00 ± 0.07, *p* = 0.7302; 7wME: 0.83 ± 0.06, *p* = 0.7302, 5-HTR_3A_, 1wME: 1.29 ± 0.17, *p* = 0.2857; 3wME: 1.03 ± 0.13, *p* = 0.9048; 5wME: 0.98 ± 0.08, *p* = 1.000; 7wME: 0.78 ± 0.06, *p* = 0.1905, Mann-Whitney test; Kruskal-Wallis test, 5-HTR_1A_: *p* = 0.2493; 5-HTR_3A_: *p* = 0.1395, Fig. 4a, b). In the Bz on the other hand, 1wME and 3wME mice showed increased 5-HTR_1A_ expression levels **(**mean ± SEM, 5-HTR_1A_, 1wME: 1.69 ± 0.12, *p* = 0.0317; 3wME: 1.44 ± 0.06, *p* = 0.0317; 5wME: 1.12 ± 0.06, *p* = 0.2857; 7wME: 0.99 ± 0.02, *p* = 0.9048, Mann-Whitney test, Fig. 4c) and a gradual decrease in 5-HTR_1A_ expression between 1wME and 7wME mice (Kruskal-Wallis test: *p* = 0.0039). In addition, 5-HTR_3A_ expression levels were significantly increased in the Bz of the 3wME mice compared to the AMCs **(**mean ± SEM, 5-HTR_3A_, 1wME: 1.59 ± 0.17, *p* = 0.1111; 3wME: 2.10 ± 0.13, *p* = 0.0159; 5wME: 1.45 ± 0.13, *p* = 0.1111; 7wME: 0.87 ± 0.08, *p* = 0.5556, Mann-Whitney test, Fig. 4d) and decreased over time to reach the AMC level at 7 weeks post-ME (Kruskal-Wallis test: *p* = 0.0038). In S1BF, 5-HTR_1A_ expression was increased in 1wME and 3wME mice, gradually decreased to reach the AMC level at 5 weeks post-ME (Kruskal-Wallis test: *p* = 0.0039), and was again increased at 7 weeks post-ME **(**mean ± SEM, 5-HTR_1A_, 1wME: 1.84 ± 0.09, *p* = 0.0159; 3wME: 1.56 ± 0.08, *p* = 0.0159; 5wME: 1.17 ± 0.07, *p* = 0.4127; 7wME: 1.60 ± 0.12, *p* = 0.0317, Mann-Whitney test, Fig. 4e). We did not observe significant modulations of 5-HTR_3A_ protein expression in S1BF **(**mean ± SEM, 5-HTR_3A_, 1wME: 1.88 ± 0.19, *p* = 0.1905; 3wME: 2.13 ± 0.34, *p* = 0.1111; 5wME: 1.28 ± 0.13, *p* = 0.5556; 7wME: 2.26 ± 0.43, *p* = 0.1111, Mann-Whitney test; Kruskal-Wallis test: *p* = 0.1325, Fig. 4f). Taken together, these observations indicate brain region-specific alterations in vMAT2-mediated presynaptic monoamine vesicle loading, as well as post-ME recovery time-dependent modulations in 5-HTR_1A_ and 5-HTR_3A_ expression levels specifically in the Bz and in S1BF but not in the Mmz.

### 5-HTR_2A_ and 5-HTR_3A_ but not 5-HTR_1A_ antagonists suppress ME-induced cross-modal plasticity

We hypothesized that, in case 5-HT receptor function is involved in the whisker-mediated reactivation of the deprived visual cortex, the ME-induced cross-modal recruitment could be suppressed by systemic administration of the 5-HTR_1A_ antagonist WAY-100635 maleate, the 5-HTR_2A_ antagonist ketanserin tartrate, or the 5-HTR_3A_ antagonist ondansetron hydrochloride during the last 3 weeks of recovery of the Mmz in ME mice (Fig. 5a-e). As the 5-HT receptors influence the excitability of brain circuits by modulating excitatory and inhibitory neurotransmission and thus by mediating the cortical E/I balance, we expected to observe effects of these drugs on cortical activity, and ultimately on cortical plasticity [36, 54, 55]. Because it was previously established that the most pronounced impact of whisker inputs on neuronal activity involves the infragranular layers of the visual cortex, as based on the expression of neuronal activity reporter gene *zif268*, these layers were interrogated separately from the supra- and granular layers.

Suppression of the 5-HTR_1A_ receptor function by long-term administration of WAY-100635 from week 5 to 7 post-ME did not alter the neuronal activity levels reached throughout the Mmz of 7wME mice, compared to saline-injected age-matched control ME mice (Mmz, supra- and granular layers: *p* = 0.9048, infragranular layers: *p* = 0.9048, Bz, supra- and granular layers: *p* = 0.0476, infragranular layers: *p* = 0.2619, Mann-Whitney test, Fig. 5f). However, it did decrease the neuronal activity in the supra- and granular layers of the Bz **(**Mean ± SEM, supra- and granular layers, saline, Bz: 71.58 ± 1.26; Mmz: 60.14 ± 1.48, WAY-100635, Bz: 65.73 ± 1.58; Mmz: 57.95 ± 3.77, infragranular layers, saline, Bz: 71.31 ± 1.57; Mmz: 56.97 ± 1.84, WAY-100635, Bz: 67.72 ± 3.35; Mmz: 55.96 ± 2.12, Fig. 5f), indicative of Bz-specific 5-HTR_1A_ expression changes during the preceding open-eye potentiation phase.

Blocking 5-HTR_2A_ receptor function by ketanserin administration from week 5 to 7 post-ME significantly decreased the neuronal activity across all layers of the Mmz (Mmz, supra- and granular layers: *p* = 0.0476, infragranular layers: *p* = 0.0238; Bz, supra- and granular layers: *p* = 0.0952, infragranular layers: *p* = 0.2619, Mann-Whitney test, Fig. 5g) but did not induce any change in neuronal activity in the Bz **(**Mean ± SEM, supra- and granular layers, saline, Bz: 71.58 ± 1.26; Mmz: 60.14 ± 1.48, ketanserin, Bz: 66.15 ± 2.83, Mmz: 51.18 ± 3.25, infragranular layers, saline, Bz: 71.31 ± 1.57; Mmz: 56.97 ± 1.84, ketanserin, Bz: 66.18 ± 3.05; Mmz: 48.85 ± 1.46, Fig. 5g). Suppression of the 5-HTR_3A_ receptor function by administration of ondansetron only reduced the neuronal activity specifically in the supra- and granular layers of the Mmz (Mmz, supra- and granular layers: *p* = 0.0476, infragranular: *p* = 0.5476; Bz, supra- and granular layers: *p* = 0.7143, infragranular layers: *p* = 0.5476, Mann-Whitney test; Mean ± SEM, supra- and granular layers, saline, Bz: 71.58 ± 1.26; Mmz: 60.14 ± 1.48, ondansetron, Bz: 71.63 ± 1.40, Mmz: 51.95 ± 2.04, infragranular layers, saline, Bz: 71.31 ± 1.57; Mmz: 56.97 ± 1.84, ondansetron, Bz: 72.64 ± 0.94; Mmz: 54.70 ± 0.40, Fig. 5h). Taken together, these results point out that modulation of the 5-HTR_2A_ and 5-HTR_3A_ receptor function, influences the late cortical response to ME, suggesting that these receptors play an important role in the cross-modal reactivation phase of the visual cortex following ME.

Previous work has shown that ME induces increased neuronal activity in the spared sensory brain areas adjacent to the deprived visual cortex. For somatosensory cortex, the *zif268*-mRNA expression levels were found to be significantly higher in adult 7wME mice compared to AMC mice [16, 53]. In order to assess the impact of pharmacological modulation of serotonergic neurotransmission on S1BF neuronal activity, next to mapping the extent of visual cortex reactivation, we compared S1BF activity levels between saline-injected 7wME mice and 7wME mice that were treated with the specific 5-HTR_1A_, 5-HTR_2A_ or 5-HTR_3A_ antagonists.

Analysis of the *zif268*-mRNA expression levels (− 1.7-(− 1.9); 2.5–4; 1, relative to Bregma) (Fig. 6a-e) indicated that the neuronal activity levels in S1BF of WAY-100635 treated 7wME mice remained unaltered across all cortical layers compared to the saline-injected 7wME mice (S1BF, supra- and granular layers: *p* = 0.9048, infragranular layers: *p* = 0.7143, Mann-Whitney test, Fig. 6f). The mice thus displayed the expected ME-induced increase in S1BF activity, despite a suppressed 5-HTR_1A_ receptor function **(**Mean ± SEM, supra- and granular layers, saline: 64.94 ± 0.49; WAY-100635: 64.90 ± 1.76, infragranular layers, saline: 65.17 ± 0.66; WAY-100635: 66.19 ± 1.67, Fig. 6f). Treatment of 7wME mice with ketanserin **(**Mean ± SEM, supra- and granular layers, saline: 64.94 ± 0.49; ketanserin: 69.77 ± 1.13, infragranular layers, saline: 65.17 ± 0.66; ketanserin: 68.68 ± 1.63, Fig. 6g) and similarly, with ondansetron **(**Mean ± SEM, supra- and granular layers, saline: 64.94 ± 0.49; ondansetron: 69.35 ± 1.11, infragranular layers, saline: 65.17 ± 0.66; ondansetron: 71.78 ± 2.38, Fig. 6h), also did not prevent the normal ME-induced increase in S1BF activity. On the contrary, it even induced higher neuronal activity across all cortical layers of S1BF, as the *zif268*-mRNA expression levels were significantly higher in ketanserin and ondansetron treated 7wME mice compared to the saline-injected control group (ketanserin, supra- and granular layers: *p* = 0.0238, infragranular layers: *p* = 0.0476; ondansetron, supra- and granular layers: *p* = 0.0238, infragranular layers: *p* = 0.0476, Mann-Whitney test). Thus, S1BF activation seems preserved in the drug-treated mice, and even intensified as visualized with 5-HTR_2A_ antagonist ketanserin and 5-HT_3A_ antagonist ondansetron.

## Discussion

In humans, dysfunction of 5-HT signaling in the brain, is best described in mental illnesses such as depression and anxiety disorders [30, 92]. Drugs that target the serotonergic system, for instance the selective serotonin reuptake inhibitors (SSRIs), are amongst the most-often prescribed first-line treatments, predominantly because low 5-HT levels in the brain were considered to be the main cause of such diseases [93]. However, the observed low efficacy and only very late-onset mood and behavioral improvements suggest large scale, functional and structural neuroplastic changes in the brain, rather than immediate, direct effects of those drugs on their targets [94–98]. Neuropsychological illnesses thus seem more complex than first considered and may in fact be neurological disorders with an underlying plasticity deficit. From research using sensory deprivation models, it is known that monoamine system-targeting drugs like SSRIs elicit plastic adaptations in the mammalian cortex [26, 99, 100]. Evidence for such cortical plasticity brought about by long-term treatment with an SSRI was already provided ten years ago and is based upon the discovery that fluoxetine can reinstate juvenile-like ocular dominance plasticity in the rodent adult visual cortex [26]. Genes involved in synaptic plasticity and chromatin structure remodeling, excitatory/inhibitory neurotransmission, transcription factors and proteolytic enzymes that degrade the extracellular matrix were found to underlie this type of plasticity [101]. It is likely that similar plastic changes occur in human patients in order to be relieved from their neuropsychological illness and to again express a positive mood and behavior.

### Late-onset ME modulates 5-HT and vMAT2 levels in adult visual cortex

Our HPLC data imply a lowered whole tissue 5-HT concentration specifically in the visual cortex of long-term ME mice. Since 5-HT release is triggered by neuronal activity in the sensory cortex, the observed decrease in 5-HT concentration in the visual cortex of 7wME mice is possibly due to the ME-induced loss of visual input in the Mmz [102]. A similar decrease in total 5-HT levels was also observed in the deprived visual cortex of adult cats in response to retinal lesions, an animal model for age-related macular degeneration [59]. For more in depth interpretation of brain region-specific alterations in 5-HT signaling, we still depend on new methodological developments that would allow reliable longitudinal analysis of the true local 5-HT release from only 1 mm thick cortical tissue, such as the Bz or the Mmz in our mouse model. For now, predictions based on longitudinal vMAT2 expression changes allow to formulate some possibilities. Tong et al., (2011) indeed already showed that vMAT2 levels are positively correlated with the tissue concentrations of total monoamines (5-HT, dopamine and noradrenaline), as measured in human tissue by means of HPLC analysis [62]. By deduction, the significantly decreased vMAT2 protein expression already shortly after the introduction of ME, may indicate decreased 5-HT concentrations in the Mmz immediately upon the loss of all its visual input. Opposite to the Mmz, the Bz displayed only a temporary increase in vMAT2 expression from week 1 to 3 weeks post-ME, at a time when its remaining open-eye inputs become potentiated. Spared cortical territory seems to share such an early 5-HT mediated response, in relation to compensating for the sensory deficit. Indeed, a similar pattern of early increased vMAT2 expression in S1BF, could go hand in hand with a fast, initial increase in 5-HT concentration and might indicate a compensatory, cortical plasticity mechanism similar to the one described by Jitsuki et al., (2011) after only 2 days of visual deprivation in S1BF of young rats [32]. Once a new functional cortical balance is established for the spared sense, it may be able to subsequently recruit nearby sensory deprived cortical territory. In our model of acquired blindness, somatosensation indeed seems to be capable of recruiting the Mmz once fine-tuned whisker-information processing has been established in S1BF.

### 5-HTR_1A_ mediates unimodal open-eye potentiation in the Bz of the visual cortex

Cortical plasticity depends on both excitation and inhibition levels, which is determined by the distribution of excitatory and inhibitory receptors. An established E/I balance defines the stability and efficacy of neuronal circuits and is therefore required for proper processing of sensory information [36, 43, 103, 104]. A study, investigating the serotonergic regulation of the two predominant types of inhibition, respectively the short intermittent bursts of phasic inhibition and the constant long-lasting tonic inhibition, aside from its effect on the pyramidal neurons in the rat visual cortex, showed that 5-HT suppressed tonic inhibition through the 5-HTR_1A_ receptor and Protein Kinase A signaling while phasic inhibition was enhanced through 5-HTR_2A_ receptor function and CaM Kinase II [105, 106]. Clearly, alterations in 5-HT receptor expression and function can exert a major impact on the excitability of brain circuits involved in brain plasticity, as this would influence the inhibitory neurotransmission and the regulation of the cortical E/I balance [36, 54, 55].

Our observations indicate ME-induced increases in 5-HTR_1A_ receptor expression in the spared cortices, the Bz and S1BF, confirming a regionally restricted and modulatory role in maintaining cortical excitability immediately upon insult. In the Bz, the increase in 5-HTR_1A_ expression occurred at the start of the open-eye potentiation phase, pointing towards an early involvement of 5-HTR_1A_-mediated tonic inhibition, which implies the consistent activation of extra- and perisynaptic GABA_A_R_δ1_ receptors on pyramidal and inhibitory neurons by ambient GABA, to regulate the overall cortical excitability during this thalamocortical plasticity process [107]. Of note, such enhanced tonic inhibition also triggers ocular dominance plasticity in the mouse visual cortex during the critical period early in life [108].

Many studies previously reported on 5-HT-mediated induction of unimodal brain plasticity in adulthood. For example, adult rats were again susceptible for monocular deprivation-induced ocular dominance plasticity after cortical infusion of 5-HT directly into the visual cortex [37]. Also in rodents, treatment with the SSRI fluoxetine, was found to induce both hippocampal synaptic plasticity and unimodal ocular dominance plasticity in the visual cortex [26, 37, 109, 110]. In line with this, in the human visual system, long-term systemic treatment with the SSRI sertraline was shown to induce synaptic plasticity by increasing the amplitude of stimulus-induced visually evoked potentials [111]. Fluoxetine even emerged as a possible treatment for persistent amblyopia, or lazy eye, in adult humans [112, 113]. Maya-Vetencourt et al., (2011) could previously also show that the ocular dominance shift, normally occurring in the Bz shortly after monocular deprivation, can be prevented by blocking this receptor with WAY-100635 [37]. Furthermore, local application of this same selective 5-HTR_1A_ antagonist in V1 by means of reverse-phase microdialysis was found to facilitate long-term potentiation (LTP) after theta-burst stimulation (TBS) in adult rats, while LTP was inhibited in juvenile rats, confirming an age-dependent role for 5-HTR_1A_ in gating V1 plasticity [27].

We were not surprised by the observation that 5-HTR_1A_ expression levels remained close to the AMC level in the Mmz, as our lab previously described that well-defined levels of GABA_A_R_α1_-mediated phasic inhibition, but not GABA_A_R_δ1_-mediated tonic inhibition, are crucial for the establishment of the cross-modal reactivation of the Mmz in adult ME mice [53]. Our interpretation, that 5-HTR_1A_ is of central importance to ME-induced open-eye potentiation in the Bz but not to the late cross-modal plasticity phase in the Mmz, is further supported by the observed downregulation of 5-HTR_1A_ receptors in the Bz from the moment that open-eye potentiation is completed, and by the absence of suppressive drug-effects in the Mmz itself during the cross-modal phase. Indeed, the normal *zif268*-mRNA expression levels observed across the upper and lower cortical layers of the Mmz argue against a substantial contribution of this receptor subtype to the cross-modal take-over of the deprived cortical territory.

### Antagonism of 5-HTR_2A_ and 5-HTR_3A_ to spatiotemporally control cross-modal brain plasticity

In this study, next to WAY-100635, we also chronically injected ketanserin or ondansetron to respectively block 5-HTR_2A_ and 5-HTR_3A_ receptor function during the entire time window of cross-modal plasticity, and following a normal period of open-eye potentiation. Although applying a local slow drug-release approach might have allowed a better discrimination of true cortical events from for example subcortical contributions to the observed plasticity phenomena, we still chose the i.p. injection approach because this systemic approach holds a greater prediction value with regards to human clinical trials than an invasive cortical infusion system. Also, long-term cannula implantation may have led to cortical tissue damage and glial scar formation. Such side-effects would have influenced the local neuromodulator levels [63] hampering the read-out as well as the interpretation of the results.

By blocking the 5-HTR_2A_ receptor with ketanserin, we successfully suppressed adult cross-modal visual cortex reactivation across all layers of the Mmz. This observation accords with literature describing a role for 5-HTR_2A_ in compensatory cross-modal plasticity [32] and with our previous observations, that post-ME cross-modal reactivation of the Mmz only occurs under well-defined levels of GABA_A_R_α1_-mediated phasic inhibition [53], which is considered to be strictly regulated by upstream 5-HTR_2A_ function [105].

5-HTR_3A_ interneurons constitute a heterogenous population. One type of interneurons dependent on 5-HT signaling are the Vasoactive Intestinal Peptide (VIP)-expressing 5-HTR_3A_ interneurons. VIP-mediated disinhibition is a well-described mechanism in which VIP interneurons inhibit somatostatin and parvalbumin expressing interneurons and may underlie ME-induced cortical plasticity [44, 46–48, 114, 115]. VIP interneurons exert an important function in controlling sensory processing and are target cells of long-range projections that integrate sensory information originating from different brain regions [116, 117]. This interneuron type thus constitutes an ideal candidate for 5-HT to shape cross-modal brain circuits and plasticity patterns via the 5-HTR_3A_ receptor. In a recent study [4], convergence of sensory and neuromodulatory information onto 5-HTR_3A_ cells was shown to vastly contribute to the shaping of critical period plasticity in the primary auditory cortex of the mouse. This research group discovered that a topographic map is formed already early in life, regulated by the non-VIP expressing 5-HTR_3A_ cell population in cortical layer I. As predicted based on the 5-HTR_3A_ cortical distribution pattern, which is predominantly in L1 of the cortex [4], our results indeed indicate that 5-HTR_3A_ receptor antagonism with ondansetron during the cross-modal reactivation phase, specifically suppressed the neuronal activity in the upper layers of the Mmz. It will be interesting to further investigate how and to what extent both the VIP and non-VIP 5-HTR_3A_ cells can spatiotemporally regulate adult cross-modal plasticity as this may enable steering brain plasticity towards the desired outcome as a future therapy, being complete functional recovery of the lost primary sense. Ex-vivo and in vivo electrophysiology and fast-scan voltammetry, should allow dissecting the local molecular cascade and cellular circuit involved in the different cortical plasticity phenomena.

### A possible strategy to improve the success rate of bionic implants

The success of bionic implants in restoring sensory function depends on our understanding of how the brain responds to sensory loss. Often, by the time the neuro-electric device is implanted, the brain has already compensated for the loss of sensory input through mechanisms of cross-modal plasticity, thereby possibly impairing the cortical translation of inputs, transmitted by the device, into meaningful sensory information [118]. During the last decades, the attempt to fully restore primary sensory function received much attention, giving rise to very promising neurobionics such as the cochlear implants to restore auditory function [119–121], visual prostheses [122–126] and brain-computer interfaces [127–129]. In addition, patients suffering from progressive vision loss, as caused by the neurodegenerative diseases *Retinitis pigmentosa* or age-related macular degeneration, can nowadays benefit from the recently developed, fully organic, subretinal prosthesis [130], which tackles some of the common mechanical, manufacturing and technical difficulties generally occurring in epiretinal [131], subretinal [132] and suprachoroidal [133] prosthetics.

Given the fact that 5-HT modulates sensory information processing and integration [134] and based on our findings that chronic, systemic administration of the 5-HTR_2A_ antagonist ketanserin and 5-HTR_3A_ antagonist ondansetron can suppress the cross-modal reorganization after partial vision loss in a cortical brain region- and layer-specific manner, we predict that a pharmacological monoamine-oriented strategy in combination with a bionic implant may significantly increase their success rate, as this approach would better allow to spatiotemporally control different forms of cortical plasticity that precede and co-occur with its functional integration.

## Conclusions

We report ME-induced molecular and functional adaptations in the visual and somatosensory cortex of adult mice. We observed brain region-specific changes in 5-HT levels, presynaptic vMAT2 expression and postsynaptic 5-HT receptor levels in function of post-ME recovery time. We showed that 5-HTR_2A_ and 5-HTR_3A_ are involved in the cross-modal reactivation of the deprived visual cortex, as pharmacological antagonism of the 5-HTR_2A_ and 5-HTR_3A_ but not 5-HTR_1A_ receptor function hampered this plasticity process, specifically at the time when whiskers normally recruit the Mmz in adult ME mice. Our findings significantly add to the current understanding of the brain plasticity phenomena, occurring in response to chronic treatment with 5-HT-system targeting drugs. In future, promising strategies to restore primary sensory functions, might become spatiotemporally controllable through 5-HT-assisted neurobionics.

## Abbreviations

5-HT: 5-Hydroxytryptamine, serotonin
5-HTR: serotonin receptor
7wME: 7 weeks post-ME
AMC: age-matched control
AMPAR1: alpha-amino-3-hydroxy-5-methyl-4-isoxazolepropionic acid receptor
BDNF: brain derived neurotrophic factor
Bz: binocular zone
E/I: excitation/inhibition balance
GABA_A_R_α1_: Gamma-aminobutyric acid receptor subunit alpha 1
HPLC: high performance liquid chromatography
i.p.: intraperitoneal
ISH: in situ hybridization
LTP: long-term potentiation
ME: monocular enucleation
Mmz: medial monocular zone
S1BF: primary somatosensory barrel field
SSRI: selective serotonin reuptake inhibitor
TBS: theta-burst stimulation
TPS: total protein stain
VIP: vasoactive intestinal peptide
vMAT2: vesicular monoamine transporter 2
WB: Western blotting
